# Targeted therapy for HER2 positive breast cancer

**DOI:** 10.1186/1756-8722-6-38

**Published:** 2013-06-03

**Authors:** Jason A Incorvati, Shilpan Shah, Ying Mu, Janice Lu

**Affiliations:** 1 Department of Medicine, State University of New York at Stony Brook Medical Center, Stony Brook, NY, USA; 2 Breast Center, Shijitan Hospital, Capital Medical University, Beijing, China

**Keywords:** HER2 positive, Breast cancer, ASCO 2012, San Antonio 2012

## Abstract

**Introduction:**

Breast cancer is the second most common cause of death for women behind lung cancer and the most common cause of cancer deaths for women aged 45–55 years old (CDC.gov 2012). Although there continue to be enormously large numbers of disease incidence, deaths have been declining due to the disease with two hallmark time frames. The first occurred during the mid to late 1980’s when hormonal therapy was introduced as a treatment for ER/PR positive breast cancer. The second occurred in the late 1990’s when trastuzumab was introduced in treating HER2 positive breast cancer. These remarkable accomplishments in developing novel targeted therapies for breast cancer, along with a better understanding of the disease biology have improved disease outcome over the past 20 years.

This article reviews the data presented at 2012 American Society of Clinical Oncology and 2012 San Antonio Breast Cancer Symposium regarding progress made in the field of HER2 positive breast cancer and examines the future of HER2 targeted therapy.

## HER2 Overview

The HER2 gene (ErbB2) is a member of a group of epithelial tyrosine kinase receptors [1,2]. These receptors also include HER1 (which is an EGFR receptor), HER3 (ErbB3), and HER4 (ErbB4) [2]. The HER2 protein tends to dimerize with either HER1, HER3, or HER4 [2].

HER2 has no identified ligand, which allows it to always be in open confirmation to dimerize with HER1, HER3, or HER4 [3]. Thus, when the HER2 gene is amplified and overexpressed, it allows for cell growth, survival, and cell differentiation through a signal transduction cascade mediated by the activation of PI3K/Akt and the Ras/Raf/MEK/MAPK pathways [4]. Before HER2 directed therapies were created, having HER2 positive breast cancer meant hyperactivation of this downstream pathway, which caused high recurrence rates and increased mortality [4-8]. The development of targeted HER2 therapies, has significantly improved the outcome for patients with HER2 positive breast cancer.

### Trastuzumab

Briefly, trastuzumab is a humanized monoclonal antibody directed against the extracellular domain of the HER2 receptor which prevents ligand-independent HER2 signaling. It was initially approved by the FDA for metastatic breast cancer in 1998 [9].

The optimal duration of adjuvant trastuzumab therapy remains an important issue. HERA (BIG 01–01) trial was presented at 2012 San Antonio Breast Cancer Symposium and published by Goldhirsch et al. [10]. The trial was a phase III randomized trial involving 5102 women with HER2 positive early breast cancer to trastuzumab (T) every 3 weeks for 1 yr, trastuzumab (T) for 2 years, or observation [10]. The unadjusted hazard ratio (HR) for an event in the 2-yr vs. 1-yr (T) arms was 0.99 (95% CI 0.85–1.14; p = 0.86) [10]. Overall survival (OS) in the two arms was comparable [HR = 1.05 (95% CI 0.86–1.28; p = 0.63)] [10]. The primary cardiac endpoint (symptomatic congestive heart failure) was comparable (1.0% vs. 0.8% for 2-yr and 1-yr arms), but the secondary cardiac endpoint (asymptomatic cardiac dysfunction) was higher in the 2-yr arm (7.2% vs. 4.1%) illustrating that at this time the standard of care of 1 year of trastuzumab therapy should remain because of the increased cardio-toxic events of longer trastuzumab therapy along with the lack of support for any additional patient benefit in the 2-yr treatment arm [10].

By contrast, the PHARE trial was designed to evaluate the advantages of shorter duration of adjuvant trastuzumab therapy. The data was supported by the results of the FINHER trial using trastuzumab for a shorter duration. The FINHER trial showed that 9 weeks of trastuzumab (T) therapy provided a similar magnitude of benefit than the standard of care 1-year treatment [11]. Thus, the French National Cancer Institute (INCa) initiated a randomized non-inferiority trial aiming to compare a shorter (T) exposure of 6 months versus the standard 12 months. 3382 patients were randomized to 6 or 12 months of (T). Unfortunately, the results were inconclusive regarding the non-inferiority (p = 0.14) [11].

Information about a newer trial, presented at the San Antonio Breast Conference, is the phase III randomized, controlled PERSEPHONE study. This trial, like the PHARE study, is comparing six months of trastuzumab to the standard 12 month duration in patients with HER2 positive early breast cancer in respect of disease free survival, safety and cost-effectiveness [12]. The goal for this trial is to evaluate patients with early stage HER2 positive breast cancer in a total of 4000 patients divided into each of the two treatment groups [12]. This trial will provide direction on the optimal duration of trastuzumab adjuvant treatment for HER2 positive early stage breast cancer.

Lastly, Romond et al. presented the final joint analysis of overall survival from the NCCTG N9831 and NSABP (B31). This study was based on a 2 arm trial of 2,028 women receiving doxorubicin/cyclophosphamide followed by paclitaxel compared to 2018 women receiving doxorubicin/cyclophosphamide followed by paclitaxel plus trastuzumab [13]. At 10 years, the disease-free survival was 73.7% with doxorubicin/cyclophosphamide followed by paclitaxel plus trastuzumab vs 62.2% with doxorubicin/cyclophosphamide followed by paclitaxel for an absolute improvement of 11.5% and 40% risk reduction (p < 0.0001) mostly related to the higher distant recurrences that occurred in the non-trastuzumab arm [13]. Similarly, the overall survival at 10 years was 84% vs 75.2% for an absolute improvement of 8.8% and 37% risk reduction (p < 0.0001) [13].

### Lapatinib

Lapatinib is a dual EGFR/ErbB2 reversible tyrosine kinase inhibitor (blocking both HER1 & HER2); thereby suppressing downstream pathways of MAPK/Erk1/2 and PI3K/Akt pathways [14]. This was the second HER2 targeted agent approved based on the study published by Geyer et al., which demonstrated that lapatinib plus capecitabine was superior to capecitabine alone in women with HER2 positive advanced breast cancer that has progressed after treatment with regimens that included trastuzumab and chemotherapy agents [15].

At ASCO 2012, the NSABP (B-41) protocol was presented by Robidoux et al. It is a randomized phase III trial of neoadjuvant therapy (anthracycline plus cyclophosphamide (AC) followed by weekly paclitaxel (WP) for patients with palpable and operable HER2 positive breast cancer comparing the combination of trastuzumab (T) plus lapatinib (L) to trastuzumab and to lapatinib administered with weekly paclitaxel following (AC) [16]. There were 529 patients randomized in the trial. The pathologic complete response (pCR) was 52.5% for AC + WP + T, 53.2% (p = 0.9 T vs L) for AC + WP + L, and 62% (p = 0.095 T vs T + L) for AC + WP + TL [16]. pCR percentages in the hormone receptor positive (HR+) subset were 46.7%, 48% (p = 0.85 T vs L), and 55.6% (p = 0.18 T vs T + L), respectively, and were 65.5%, 60.6% (p = 0.57 T vs L), and 73% (p = 0.37 T vs T + L) in the hormone receptor negative (HR-) cohort [16]. The corresponding pCR breast and nodes percentages were 49.1%, 47.4% (p = 0.74), and 60.4% (p = 0.04) [16]. It was concluded that substitution of lapatinib for trastuzumab in combination with the chemotherapy program employed in this study resulted in similar high percentages of pCR in both HR + and HR- cohorts [16]. Combined HER2 targeted therapy produced a numerically higher pCR percentage than single agent HER2 directed therapy, but the difference was not statistically significant [16].

Currently, Takano et al. is evaluating a randomized phase II trial (ELTOP) in patients with HER2 positive metastatic breast cancer (MBC) previously treated with trastuzumab and taxanes comparing capecitabine 2,500 mg/m^2^/day on days 1 to 14 plus trastuzumab (8 mg/kg loading dose and 6 mg/kg thereafter) on day 1 every 3 weeks (HX) and capecitabine 2,000 mg/m^2^/day on days 1 to 14 plus lapatinib 1250 mg/day on days 1 to 21 every 3 weeks (LX) [17]. The study’s primary endpoint is progression-free survival with secondary endpoints in overall response rate, overall survival, proportion of patients progressing brain metastases as site of first progression, and safety [17]. The study is also investigating biomarkers related to the HER family [17].

Other clinical data presented from the 2012 San Antonio Breast Symposium was from a phase I/II trial of primary chemotherapy with non-pegylated liposomal doxorubicin, paclitaxel and lapatinib in patients with HER2 positive early stage breast cancer (P1-14-05) [18]. A total of 84 patients were included in the trial [18]. Preliminary data reveals the combination of non-pegylated liposomal doxorubicin, paclitaxel and lapatinib is well tolerated with no worsening cardiac toxicity and has high antitumor activity in patients with HER2 positive primary breast cancer [18].

A subsequent study by Kodack et al. (P3-12-03), is looking at combined targeted therapy with HER2 and VEGFR2 for effective treatment of HER2 amplified breast cancer brain metastases [19]. Typically, brain metastases do not respond well to HER2 inhibitors and are often the reason for treatment failure [19]. Pre-clinical data with an HER2 amplified mouse model of brain metastasis using an orthotopic xenograft of BT474 cells in mice, has revealed that the combination of either trastuzumab and DC101 (anti-VEGFR2 inhibitor) or lapatinib and DC101 significantly slowed metastatic tumor growth in the brain with marked necrosis of brain lesions. This resulted in a striking improvement in overall survival in mice [19]. Thus, a new clinical trial is now recruiting patients to evaluate the efficacy of bevacizumab in breast cancer patients with active brain metastases, including its combination with trastuzumab in patients with HER2 positive disease [19].

The MA.31 trial was also presented at ASCO 2012. This trial tested the relative efficacy of lapatinib (L) vs trastuzumab (T) in first line metastatic breast cancer. The study accrued 652 patients. The regimen of arm 1 was (L) dose of 1250 mg with Tax followed by 1500 mg (L) daily (LTax/L) compared to arm 2 of (T) 2 mg/kg weekly or 6 mg/kg every 3 weeks plus Tax followed by (T) 6 mg/kg every 3 weeks (TTax/T) [20]. The progression free survival was inferior with (LTax/L) of 8.8 months compared to (TTax/T) of 11.4 months [20]. There was no difference in overall survival between the 2 arms [20]. Thus, it appears that lapatinib is associated with a shorter progression free survival as a first line treatment in metastatic breast cancer patients compared to trastuzumab.

The San Antonio Breast Conference provided one more report on Lapatinib investigative trials, Estevez et al. (P5-18-15) investigated lapatinib in HER2 positive ductal carcinoma in situ (DCIS) [21]. The study was aimed at determining the effects of Ras/Raf/MAK and PI3K/AKT signaling pathway of the HER2 inhibitor lapatinib in patients with HER2 positive DCIS, as well as the correlation with radiological and pathological responses [21]. A total of 20 patients with HER2 positive DCIS received a dose of 1500 mg daily of lapatinib for 4 consecutive weeks prior to surgical resection with radiographic changes evaluated by MRI [21]. The results revealed that lapatinib modulated HER2 signaling in 11 patients by decreasing cytoplasm pERK in 11/20 patients and 7/20 presented with decrease in MRI signaling and tumor size [21]. Thus, Esteves concluded that 4 weeks of pre-surgical lapatinib in DCIS revealed significant anti-proliferative effects [21].

### Synergy with lapatinib and trastuzumab

With newer HER2 drugs in the pipeline, researchers investigated the effects of using lapatinib and trastuzumab together. It has been found that lapatinib could enhance trastuzumab by increasing the apoptotic effect [4]. Blackwell et al. presented results of the Phase III EGF104900 trial, which demonstrated that lapatinib plus trastuzumab significantly improved progression-free survival (PFS) and clinical benefit rate versus lapatinib monotherapy [22]. A total of 291 randomly assigned HER2 positive metastatic breast cancer patients whose disease that progress during prior trastuzumab therapy were assigned to receive lapatinib monotherapy or a combination of lapatinib and trastuzumab [22]. The study concluded that absolute overall survival (OS) rates were 10% at 6 months and 15% at 12 months in the combination arm (lapatinib/trastuzumab) compared with the monotherapy (lapatinib) arm demonstrating a significant 4.5-month median OS advantage with lapatinib and trastuzumab combination in support dual HER2 blockade in patients previously treated HER2 positive metastatic breast cancer [22].

At the San Antonio Breast Conference 2012, Johnson et al. presented the ALTERNATIVE Study, which is a phase III, randomized, open-label, multicenter trial examining the efficacy of lapatinib/trastuzumab/aromatase inhibitor versus trastuzumab/aromatase inhibitor [23]. The theory regarding this trial is the fact that HER2 positive patients is associated with a poor prognosis and hormone resistance to endocrine therapy in patients that are hormone receptor positive; thus, this trial wants to evaluate how dual anti-tyrosine kinase inhibitor with an aromatase inhibitor compares to single agent tyrosine kinase with an aromatase inhibitor in overall survival. The target date for initial results is in 2016 [23].

Crown et al. also presented an ongoing phase III study evaluating dual anti tyrosine kinase, however, this study is looking at hormone receptor negative breast cancer with the combination of paclitaxel in the first line treatment of metastatic breast cancer [24]. This study will not only evaluate for efficacy but also predictive biomarkers of response to trastuzumab and or lapatinib [24]. In similar fashion, Lin et al. is evaluating whether the combination of trastuzumab and lapatinib in metastatic breast cancer undergoing maintenance therapy as well improves progression free survival [25].

### Newer HER2 drug therapies

In addition to HER2, HER1 and particularly HER3, play an important role in forming heterodimers with HER2 allowing for critical activation of the PI3K/Akt pathway driving the pathways of angiogenesis, cell survival, migration, apoptosis, and cell proliferation [26]. The drug therapies are summarized in Table 1.

**Table 1:** Summary of drug therapies

**Target**	**Agent**	**Most common adverse events reported to FDA**	**Relevant protocol(s)**	**Pharmaceutical source**
Heterodimerization of HER2 with HER3 receptor	Pertuzumab	Diarrhea, alopecia, neutropenia, nausea, fatigue, rash, peripheral neuropathy, infusion and hypersensitivity reactions	CLEOPATRA (NCT00567190) NCT01358877	Roche-Genentech
			VELVET (NCT01565083)	
			PERTAIN (NCT01491737)	
			PERUSE (NCT01572038)	
HER2 receptor	Trastuzumab-maytansine [DM1]	Fatigue, nausea, thrombocytopenia, cellulitis, elevated liver enzymes, left ventricular dysfunction, neurotoxicity	EMILIA (NCT00829166)	Roche-Genentech
			MARIANNE (NCT01120184)	
Multi-targeted receptor tyrosine kinase inhibitor	Pazopanib	Diarrhea, change in hair color, nausea, vomiting, loss of appetite, fatigue, liver dysfunction	VEG108838 (NCT00558103)	GlaxoSmithKline
			NSABP (FB-6) (NCT00849472)	
Irreversible binder of the HER receptors [HER1, HER2, and HER3]	Afatinib	Safety and efficacy not fully established by the FDA.	NCT00826267	Boehringer Ingelheim
		Likely gastrointestinal and skin-related side effects from HER 1 blockade	LUX-Breast 1 (NCT01125566)	
			LUX-breast 2 (NCT01271725)	
			LUX-breast 3 (NCT01441596)	
			NCT01325428	
Irreversible binder of the HER receptors [HER1, HER2, and HER3]	Neratinib	Safety and efficacy not fully established by the FDA.	ExteNet (NCT00878709)	Puma Biotechnology
			NSABP (FB-8) (NCT01423123) NCT01494662	
			NSABP FB-7 (NCT01008150)	

### Pertuzumab

Pertuzumab is a monoclonal antibody which attempts to block the heterodimerization of HER2 with HER3 by interfering with the ligand-dependent HER3 mediated signaling [26]. This drug has only shown modest antitumor clinical activity alone; however, it appears to be a very good synergistic drug combined with trastuzumab. The NEOSPHERE, neoadjuvant trial, took patients with operable HER2 positive breast cancer (n = 417) and placed them in four arms receiving 4 cycles: docetaxel plus trastuzumab and pertuzumab, docetaxel plus trastuzumab, docetaxel plus pertuzumab, or pertuzumab plus trastuzumab without chemotherapy [27]. The pathologic complete response rates were 45.8% for the combined regimen of docetaxel plus trastuzumab + pertuzumab, 29% in docetaxel plus trastuzumab (P = 0.014), 24% in docetaxel plus pertuzumab (P = 0.003), and 16.8% in the trastuzumab + pertuzumab without chemotherapy [27]. This study led to the CLEOPATRA study, which takes HER2 positive metastatic breast cancer patients randomized to pertuzumab + trastuzumab and docetaxel or placebo + trastuzumab and docetaxel [28]. In the CLEOPATRA study, 808 patients with HER2 positive metastatic breast cancer were randomly assigned with the primary end point being progression-free survival [28]. The median progression-free survival was 18.5 months in the pertuzumab group compared to 12.4 months in the control group, which led to the FDA approval of Pertuzumab [28]. In the intention-to-treat population, 267 patients died by data cutoff (May 14, 2012), 154 (38%) of 406 in the placebo group and 113 (28%) of 402 in the pertuzumab group [29]. Median overall survival was 37.6 months (95% CI 34.3—NE [not estimable]) in the placebo group but had not been reached (95% CI 42.4—NE) in the pertuzumab group (hazard ratio 0.66, 95% CI 0.52-0.84; p = 0.0008) [29].

Pertuzumab is being evaluated in a phase III study (ClinicalTrials.gov identifier: NCT01358877 “APHINITY”) as an adjuvant therapy in order to investigate the optimal regimen for HER2 positive breast cancer with the primary outcome being disease free survival [30]. Patients will be randomized (1:1) prior to any chemotherapy to one of 2 treatment arms – standard anthracycline-based chemotherapy for 3–4 cycles followed by taxane × 3–4 cycles with trastuzumab with or without pertuzumab (for a total of 52 weeks). Another approach is to give non-anthracycline-based chemotherapy (docetaxel + carboplatin) × 3–4 cycles followed by trastuzumab with or without pertuzumab for 52 weeks [30].

A phase II study, VELVET trial, by Perez et al. is an ongoing trial evaluating combination of pertuzumab, trastuzumab and vinorelbine for first-line treatment in patients with HER2-positive locally-advanced or metastatic breast cancer to assess response rate and safety is also ongoing [31]. This trial will provide input regarding the use of pertuzumab and trastuzumab with chemotherapy besides taxane.

Another ongoing phase II study, PERTAIN trial, by Rimawi et al. is comparing trastuzumab and a aromatase inhibitor with or without pertuzumab as first-line therapy in hormone-positive, HER2 positive metastatic breast cancer in postmenopausal women [32]. Patients can receive induction chemotherapy (taxane) as per investigator’s discretion. This trial will provide insight into the benefits of single or dual HER2 targeted therapies in combination with aromatase inhibitor.

At the 2012 San Antonio Breast Conference, Baselga and colleagues presented an analysis which explored the predictive and/or prognostic value of biomarkers in tissue samples and serum in the CLEOPATRA study population [33]. These markers included HER1, HER2, HER3, PTEN, pAKT, AREG, c-myc, betacellulin etc. detected by methods such as immunohistochemistry, reverse transcription polymerase chain reaction, ELISA and fluorescence *in situ* hybridization [33]. The results suggested that HER2 remains the only marker suitable for use when selecting patients for anti-HER2 therapy. There was an indication that the presence of the PI3K mutation would suggest a poorer prognosis within the HER2 positive population, and that perhaps adding a PI3K pathway-targeted agent would improve outcomes [33].

Another subgroup analysis by Miles and colleagues evaluated the effect of age in the CLEOPATRA study comparing the safety and efficacy in elderly patients [34]. The conclusion from the adverse effect (AE) profile was that age should not restrict the use of Pertuzumab.

Two ongoing trials were presented at the San Antonio Breast Symposium, which looked at pertuzumab. The first was a single arm phase IIIb study of pertuzumab and trastuzumab with a taxane as first-line therapy for patients with HER2 positive advanced breast cancer (PERUSE) [35]. PERUSE will evaluate patients with HER2 positive metastatic or locally recurrent breast cancer who have not received systemic therapy for metastatic cancer to assess the safety and tolerability of trastuzumab + pertuzumab with a choice of taxane as first-line therapy [35]. The second was a prospective, phase II study evaluating the therapeutic value of liposomal anthracyclines in HER2 positive breast cancer with dual anti-HER2 therapy (trastuzumab + pertuzumab) in evaluating cardiac safety given the possibility of increased cardiotoxicity with anthracyclines and trastuzumab [36].

### Ttrastuzumab-maytansine [dm1]

Trastuzumab-maytansine [DM1] is an immunoconjugate agent combining trastuzumab with an antimicrotubule cytotoxic chemotherapeutic agent linked together by a covalent bond [26]. In Phase I and Phase II studies the drug has been found to be well-tolerated with significant objective response rates and improvements of progression-free survival.

Hurvitz et al. presented phase II data at ESMO 2011 and the data was subsequently published in 2013 [37]. The study showed that the patients treated with trastuzumab emtansine (T-DM1) achieved roughly a 40% reduction in risk of disease progression compared with those on a standard combination of trastuzumab plus docetaxel (14.2 months versus 9.2 months), (HR 0.59, *P* = 0.035) [37]. A total of 137 patients with locally advanced or metastatic HER2 positive breast cancer naive to chemotherapy or targeted therapy were examined with a primary endpoint of progression-free survival [37]. Overall, the toxicity was far lower among those on the investigational agent (46.4% versus 89.4%) and more women on standard care discontinued therapy due to side effects (28.8% versus 7.2%) [37]. Thus, it was concluded that first-line treatment with T-DM1 for patients with HER2 positive metastatic breast cancer provided a significant improvement in PFS, with a favorable safety profile, compared to standard combination therapy of trastuzumab plus docetaxel.

EMILIA is a T-DM1 registration trial comparing trastuzumab-DM1 versus lapatinib plus capecitabine, which led to FDA approval of this drug in February of 2013 [38]. The primary results from this phase III study presented by Blackwell et al., in HER2 positive locally-advanced or metastatic breast cancer patients previously treated with trastuzumab and taxane examined 978 patients with the primary endpoint being PFS, OS and safety [38]. Blackwell reported that there was a significant improvement in median progression-free survival 9.6 months in the T-DM1 arm compared with 6.4 months in the capecitabine/ lapatinib arm (stratified hazard ratio [HR] = 0.650, p < 0.0001) and safety and secondary efficacy endpoints favored T-DM1 [38]. These result led to the approval of T-DM1 in February of 2013. The final overall survival analysis will be fully presented in 2014; however, preliminary data reveals that there is a trend for improvement in overall survival 84.1% with T-DM1 compared to 77% with capecitabine/lapatinib at 1 year and 65.4% with T-DM1 and 47.5% with capecitabine/lapatinib at 2 years [38].

The MARIANNE trial is a phase III 3-arm study evaluating TDM-1, TDM-1 plus pertuzumab or trastuzumab plus a taxane as first line metastatic regimen [39]. The result will provide insight regarding which regimen is the winner for first line metastatic breast cancer.

At the San Antonio Breast Conference, Wang et al. presented an evaluation of the pharmacokinetics and exposure-efficacy relationship of T-DM1 in the EMILIA phase III study. Wang concluded that T-DM1 pharmacokinetics data were similar in previous phase II studies with historical data of single agent T-DM1 [40]. Furthermore, there was no significant exposure-efficacy relationship observed between T-DM1 AUC (*P* = 0.23 for PFS and P = 0.53 for OS) or DM1 Coax (*P* = 0.96 for PFS and *P* = 0.34 for OS) and PFS or OS, respectively [40].

### Pazopanib

Pazopanib is a selective multi-targeted receptor tyrosine kinase inhibitor of VEGF receptors 1, 2, 3, PDGF receptor a/β, and cytokine receptor c-kit that blocks tumor growth and inhibits angiogenesis. At ASCO 2012, a phase II study comparing lapatinib (1500 mg) plus placebo and lapatinib (1500 mg) with pazopanib (800 mg) in patients with relapsed HER2 positive inflammatory breast cancer (IBC) was presented by Cristofanilli et al. [41]. 76 patients were examined in a 1:1 double blind study until disease progression, unacceptable toxicity, or death occurred. Due to significantly increased incidence of grades 3 and 4 diarrhea at this dose (71% in the combination arm versus 24% in lapatinib arm) this cohort was closed [41]. Thus, the protocol was amended such that an additional 88 patients were randomized in a 5:5:2 ratio to receive daily monotherapy lapatinib 1,500 mg, lapatinib 1,000 mg + pazopanib 400 mg, or monotherapy pazopanib 800 mg with a primary endpoint of obtaining an overall response rate (ORR) [41]. In this amended cohort, the ORR for patients treated with lapatinib (n = 36) was 47%, lapatinib + pazopanib (n = 38) was 58%, and pazopanib (n = 13) was 31%. However, the median PFS was not increased compared to lapatinib alone [41]. The combination also had increased toxicity resulting in more dose reductions, modifications, and treatment delays [41]. It was concluded that there was no clinical benefit of the combination as opposed to single agent lapatinib [41]. In fact, there was increased incidence of toxicities with the combination especially at higher dose of pazopanib.

### Afatinib

Afatinib is an irreversible binder of the HER receptors [HER1, HER2, and HER3] [26]. The drug appears to have anti-tumor activity by itself or with chemotherapy in patients with progressive HER2 positive breast cancer. As presented at ASCO 2012, a small phase II trial studying single-agents afatinib (50 mg daily), lapatinib (1500 mg daily) and trastuzumab (2 mg/kg weekly after loading dose) as neoadjuvant monotherapy in locally-advanced (stage III and inflammatory BC) for 6 weeks pre-operatively suggested that afatinib had higher objective response rate compared to the other 2 agents (80% vs. 75% vs. 36% respectively) [42]. It was concluded that further studies are needed to identify the potential role of afatinib as a single-agent or in combination in HER2 positive breast cancer patients.

To assist in addressing this issue, there is an ongoing phase III study of patients, previously treated with trastuzumab, which is evaluating vinorelbine plus afatinib versus vinorelbine plus trastuzumab [43]. The Phase III, open-label randomized study began enrolling in June 2010 with a recruitment target of 780 patients and a primary end-point being PFS [44]. The patients will be randomized in a 2:1 fashion between Afatinib + Vinorelbine and Trastuzumab + Vinorelbine arms [44]. The dose of afatinib is 40 mg PO daily, vinorelbine is 25 mg/m2/wk and trastuzumab is 2 mg/kg/week after initial loading dose of 4 mg/kg. Patients will receive continuous treatment until disease progression or toxicity [44].

Besides this phase III trial, there are three other phase II studies that were presented.

Firstly, Hickish et al. presented a phase II study (LUX-breast 2) evaluating the objective response of afatinib at dose of 40 mg/day in metastatic breast cancer patients who progressed on prior trastuzumab or lapatinib as single-agent followed by afatinib ‘beyond progression’ with chemotherapy (weekly Paclitaxel 80 mg/m2 or Vinorelbine 25 mg/m2) [45]. 120 patients who do not have rapidly progressing visceral disease or active brain metastasis will be evaluated [45]. Enrollment began in May 2011 at about 35 sites in 5 countries with a primary endpoint being objective response [45].

Secondly, LUX-breast 3 trial presented by Joensuu et al. is an ongoing phase II study, which randomizes HER2 positive breast cancer patients with progressive brain metastases (after trastuzumab or lapatinib based therapy) to single-agent afatinib versus afatinib + vinorelbine versus the investigator’s choice of approved therapy [46]. The study began in Oct 2011 and is still enrolling. The primary end-point is benefit at 12 weeks (absence of CNS/ extra-CNS disease progression and no tumor-related neurological worsening with any increase in steroid dose) [46].

Lastly, Swanton et al. presented a phase II trial evaluating the efficacy and safety of afatinib in inflammatory breast cancer [47]. This study takes into account the fact that inflammatory breast cancer commonly lacks ER/PR and more frequently harbors HER2 gene amplification (~40%), and EGFR overexpression (~30%), which is associated with a poor prognosis [47]. Thus, patients with HER2 expressing inflammatory breast cancer will be given afatinib (that binds to HER1 & HER2) with or without vinorelbine. Both trastuzumab naïve and failed patients are being enrolled since Aug 2011 with a primary end-point of Clinical Benefit Rate (CR, PR or SD) for greater than 6 months [47].

### Neratinib

Neratinib, which is similar to Afatinib, is another irreversible binder of the HER receptors [HER1, HER2, and HER3] [26]. This drug has been looked at in studies with patients that are HER2 positive and have been exposed to prior trastuzumab treatment or never have received any anti-HER2 treatment in the past. In phase II studies, neratinib has been shown to be well-tolerated and shown significant clinical activity [48]. A phase II study looking at 136 HER2 positive patients showed a 24% response rate in women previously treated with trastuzumab with a progression-free survival at 16 weeks of 59%, and a 56% response rate in patients who have never received any anti-HER2 treatment prior with a progression-free survival of 78% at 16 weeks [49]. It has also been shown to be more potent and have a more prolonged inhibitory effect as compared to lapatinib and can bypass potential resistance pathways [49]. These positive results have led to several phase III studies with neratinib. For example, the EXTENET trial is a randomized, double-blind, placebo-controlled trial of neratinib after trastuzumab in women with early-stage HER2 overexpressed/amplified breast cancer [50]. The purpose of this study is to investigate whether neratinib can further reduce the risk of recurrence from previously diagnosed HER-2 positive breast cancer after adjuvant treatment with trastuzumab, and evaluate for disease-free survival as the primary endpoint with results expected by 2017 [50]. At ASCO 2012, Canonici et al. presented effect of neratinib alone and in combination with trastuzumab in HER2 positive breast cancer cell lines [51]. The results indicate that in HER2 positive cell lines, including trastuzumab and/or lapatinib resistant cells, are sensitive to neratinib alone with IC_50_ values (concentration which inhibits 50% of growth). This study showed significant benefit with neratinib as a single agent and in combination with trastuzumab in both trastuzumab sensitive and lapatinib resistant cell lines [51]. It was concluded that neratinib should be studied further in HER2 positive breast cancer patients, including trastuzumab and lapatinib resistant cases.

A phase I NSABP study (FB-8) by Jankowitz et al. evaluated weekly paclitaxel with neratinib and trastuzumab in women with metastatic HER2 positive breast cancer. It showed good tolerability and activity in patients pre-treated with anti-HER2 therapy and chemotherapy [52]. This study provided recommendations for doses of the neratinib and trastuzumab combination for phase II NSABP FB-7 trials, with the purpose of determining the activity and safety profile of Neratinib as a mono-blockade or in combination with Trastuzumab as dual blockade in neoadjuvant therapy of locally advanced breast cancer (stage IIB, III A, B and C) followed by postoperative trastuzumab for one year [52]. A total of 126 patients will be enrolled in the study with a primary goal of determining the pathologic complete response in breast and axillary lymph nodes following completion of neoadjuvant therapy. The efficacy of neratinib as a multi inhibitor of the HER family has yet to be fully proven [52].

The results of an interesting trial [TBCRC 022] presented at the San Antonio Breast Conference looked at Neratinib in CNS disease. Pre-clinical data suggests it may cross the blood brain barrier [53]. This phase II, multicenter, open-label study will evaluate patients with HER2+ breast cancer and brain metastases with a primary endpoint of CNS objective response rate (ORR) and additional endpoints including non-CNS ORR, progression-free survival, overall survival (OS), site of 1st progression, and toxicity [53]. This trial may assist in providing more drugs in the arsenal to fight CNS positive HER2 breast cancer.

## Conclusion

In this article, we reviewed studies presented at 2012 American Society of Clinical Oncology and 2012 San Antonio Breast Cancer Symposium on the development of HER2 positive breast cancer treatments. Although great strides have been made in the treatment of breast cancer and in particular, HER2 positive breast cancer, the disease has not been conquered. There are diverse potential mechanisms that make the currently FDA approved HER2 targeted agents, trastuzumab, lapatinib, pertuzumab, and T-DM1 to be refractory. One of the many challenges we struggle with are the multiple pathways of resistance involved. We seek to discover ways in which the combination therapy may assist in blocking these pathways. Many new agents, some discussed in this paper, present new and exciting strategies to combat HER2 positive breast cancer, which provide additional safe and effective treatment options, either as single agents or in combination regimens. Equally as important, further investigation is ongoing in evaluating biological markers to assist in tailoring treatments to the individual and not just to the disease. Identification of potential biomarkers would provide the clinician valuable information with regard to response and clinical benefit [54-56]. Many of the clinical trials that are currently ongoing collect tissue and blood samples and probe for potential biologic markers related to cell proliferation, cell cycles, angiogenesis, or signal transduction [54-56]. Hopefully these studies will help us further understand the mechanism of resistance and provide cure for HER2 positive breast cancer.

In order to better understand HER2 positive breast cancer, we need further dynamic and innovative clinical trial designs in the form of basic, translational, and clinical research to improve patient outcomes in drug targeted HER2 positive breast cancer. For example, T-DM1 was recently approved by the FDA as second line metastatic therapy. Can T-DM1 be given as a first line agent given its efficacy and low toxicity profile? What about evaluating dual anti-HER2 therapy, such as T-DM1 plus pertuzumab as opposed to T-DM1 alone in the neoadjuvant and adjuvant settings? How effectively does neratinib overcome resistance with combination therapy with, for instance, trastuzumab, pertuzumab or T-DM1? Such pertinent questions are being evaluated by ongoing and future trials and need to be addressed, along with many others by researchers.

We foresee that in the future, HER2 breast cancer will no longer be lumped as a single entity in breast cancer, but a multiple different subsets within a single entity of HER2 breast cancer. The ultimate purpose is to be able to customize drug therapy based on a variety of clinical and translational research, and to optimize drug treatment for HER2 positive breast cancer.

## Competing interests

The authors declare that they have no competing interests.

## Authors’ contributions

JI, SS, YM and JL drafted the manuscript. All authors read and approved the final manuscript.
